# Serologic and molecular evidence of avian metapneumovirus subtypes A and B in unvaccinated broiler breeder flocks in Egypt (2024–2025)

**DOI:** 10.1186/s12917-026-05459-y

**Published:** 2026-05-09

**Authors:** Omar S. Saeed, Mirna Akram Labib, Mahmoud Gamal, Basem M. Ahmed, Ayman H. El-Deeb, Haitham M. Amer

**Affiliations:** 1 Department of Virology, Faculty of Veterinary Medicine, Cairo University, Giza, 12211 Egypt; 2 Department of Pharmacology, Faculty of Veterinary Medicine, Cairo University, Giza, 12211 Egypt; 3 Department of Biochemistry and Molecular Biology, Faculty of Veterinary Medicine, Cairo University, Giza, 12211 Egypt; 4 Center for Biotechnology and Interdisciplinary Studies, Rensselaer Polytechnic Institute, Troy, NY USA; 5 Faculty of Veterinary Medicine, King Salman International University, El-Tor, Egypt; 6 Faculty of Veterinary Medicine, Egyptian Chinese University, Cairo, Egypt

**Keywords:** Avian metapneumovirus, Broiler breeder, Egypt, RT-qPCR, Surveillance, Subtype B

## Abstract

**Background:**

Avian metapneumovirus (aMPV) is an economically significant respiratory pathogen of poultry that affects performance, egg production, and fertility of breeder flocks. Despite its impact, aMPV continues to be ill-defined in Egypt with no integrated surveillance studies conducted in breeder flocks. Therefore, the current study was designed to provide the first integrated molecular and serological evaluation of aMPV circulation in broiler breeders in Egypt.

**Methods:**

Between 2024 and 2025, a total of 6,000 serum samples and 800 tracheal swabs were collected from 60 unvaccinated broiler breeder flocks across twelve Egyptian governorates. Sera were obtained at 16 weeks (rearing phase) and 35 weeks (production phase) of age and screened for aMPV subtypes A and B by ELISA. Tracheal swabs collected during the production phase were pooled into 80 composite samples and tested for the viral RNA by reverse transcription quantitative real time PCR (RT-qPCR).

**Results:**

Serological analysis revealed widespread aMPV exposure, with governorate-level seroprevalence ranging between 64.4% (Luxor) and 89% (Giza). Antibody titers increased between 16 and 35 weeks of age, reflecting cumulative viral exposure. Molecular testing detected aMPV RNA in 67 (83.75%) of pooled swab samples. Subtype B was the predominant genotype detected solely in 65 (81.25%) sample pools and in co-detection with subtype A in 2 pools (2.5%). Serological and molecular findings were generally aligned, with flocks positive for aMPV RNA often exhibiting higher antibody titers.

**Conclusion:**

These findings indicate that aMPV, particularly subtype B, is likely endemic across the Egyptian broiler breeder flocks. The study highlights critical knowledge gaps and emphasizes the need for viral isolation, sequencing, and controlled evaluation of biosecurity and vaccination strategies.

**Supplementary Information:**

The online version contains supplementary material available at 10.1186/s12917-026-05459-y.

## Background

Avian metapneumovirus (aMPV), formerly known as avian pneumovirus or turkey rhinotracheitis virus, is a major respiratory pathogen of poultry. The virus is associated with upper respiratory tract disease characterized by sinusitis, facial swelling, swollen head syndrome (SHS), reduced egg production, and increased mortality, resulting in significant economic losses [1, 2]. Its ability to infect multiple avian species including turkeys, chickens, guinea fowl, ducks, and pheasants highlights its broad host range and epidemiologic importance [3, 4]. Since its first identification in South African turkeys in 1978 [5–7] and its subsequent implication in SHS in chickens in England [8], aMPV has spread widely across Europe [9], Asia [10–13], Africa [14–17], the Americas [18–22], and in the Middle East region [23–29].

As a typical member of genus *Metapneumovirus* within the family *Pneumoviridae*, aMPV is an enveloped virus with helical capsid and a negative-sense single-stranded RNA genome of approximately 13.3–14 kb in length. Distinct open reading frames have been described within the viral genome to encode eight proteins including: nucleoprotein (N), phosphoprotein (P), matrix proteins (M and M2), fusion protein (F), small hydrophobic protein (SH), attachment glycoprotein (G), and the large polymerase (L). The N protein is highly conserved and is widely used in serodiagnosis, whereas G protein is highly variable and serves as a major determinant for molecular subtyping [30].

Four primary aMPV subtypes (A, B, C, and D) have been identified. The aMPV subtypes exhibit distinct geographic distributions and genetic divergence. Subtypes A and B predominate across Europe, Africa, and most of Asia, subtype C is mainly reported in North America and parts of Asia, and subtype D has been detected only in France. Despite high conservation within each subtype (e.g., subtype B isolates share ~ 97.7–99.98% nucleotide identity), substantial variation exists among subtypes. Comparative analyses of structural genes showed moderate similarity between subtypes A and B (e.g., P protein ~ 72% and M2‑1 protein ~ 89% amino acid identity), whereas subtype C was more divergent, with the M gene sharing only ~ 70.6–71.7% nucleotide identity with A or B isolates [31, 32].

The attachment (G) glycoprotein gene is highly variable, showing only ~ 25–52% identity between subtypes, reflecting marked antigenic and phylogenetic differences. Subtype D is recognized as distinct, although detailed nucleotide comparisons with other subtypes are limited in the literature [33, 34]. Recently, additional divergent aMPV-like viruses have been identified in North American wild birds, which indicates further expansion of aMPV genetic diversity [35, 36].

As a component of the multifactorial respiratory disease complex, aMPV predisposes flocks to secondary infections by *Escherichia coli*, *Mycoplasma gallisepticum*, and *Ornithobacterium rhinotracheale*, with increased morbidity and complicated diagnosis [37]. Disease severity varies with species and age, with turkeys and broiler chickens often showing more severe outcomes [38, 39]. While clinical signs may raise suspicion, definitive diagnosis requires laboratory confirmation. Virus isolation is often constrained by the short duration of viral shedding period and sample degradation, whereas molecular assays provide rapid and sensitive detection. In experimentally infected chickens, aMPV subtype B shedding typically occurs between 3–7 days post-infection, peaking around day 5, thereby limiting the window for successful isolation [4]. Real-time reverse transcription quantitative polymerase chain reaction (RT-qPCR) is considered one of the most efficient techniques for routine surveillance, early detection, and subtype differentiation [40, 41].

The pooling strategy is widely applied in molecular surveillance programs for avian respiratory viruses to optimize laboratory resources while maintaining broad flock-level coverage, particularly in large-scale epidemiological investigations. This approach enables efficient surveillance of multiple flocks across geographically distributed areas [24]. Although pooling may reduce analytical sensitivity in low-titer samples due to dilution effects, it remains an accepted strategy for flock-level surveillance. If necessary, positive pools can be subjected to follow-up individual testing to confirm intra-flock infection [42].

Serological assays are practical for population-level surveillance, where ELISA kits utilizing the conserved N protein enable reliable detection of natural aMPV exposure. Although some assays are optimized for subtypes A and B, ELISA remains essential for monitoring infection pressure, evaluating flock immunity, and guiding vaccination policies [43–46].

In Egypt, respiratory viral infections continue to challenge poultry production despite widespread vaccination against avian influenza, Newcastle disease, and infectious bronchitis [47–49]. Egypt’s poultry industry operates within a vertically integrated production system that combines large commercial enterprises with an extensive network of small- and medium-scale producers, collectively supplying most of the national animal protein demand. The broiler sector represents the largest component of this system, with commercial farms contributing approximately 90% of total chicken production between 2017 and 2023. Approximately 10 million broiler breeder (parent stock) birds form the upper tier of the production pyramid, sustaining a densely distributed broiler population primarily concentrated in the Nile Delta region and Giza governorate [50].

However, the epidemiology of aMPV remains poorly understood since most studies are either fragmented or geographically limited. Both subtypes A and B have been detected in turkeys, chickens, and ducks, often alongside bacterial co-infections [23, 51–54]. Despite these detections, comprehensive documentation of vaccination practices in commercial broiler breeder operations remains limited.

Vaccination policies appear to vary between production systems and are generally implemented under veterinary supervision rather than through a coordinated national framework. Where applied, vaccination typically involves imported live attenuated subtype A- or B-based vaccines, occasionally followed by inactivated boosters during the production phase. In addition to the imported commercial products, locally produced inactivated vaccines derived from the Egyptian strains have been developed and shown to confer protective efficacy under experimental conditions [55].

Commercial vaccines available in the Egyptian market include live subtype B vaccines (e.g., Vaxxon SHS, NEMOVAC, AVIFFA RTI, RESPIVAC aMPV), a live subtype A vaccine (Poulvac TRT), and inactivated subtype B vaccines (HIPRAVIAR TRT and TUR-3). Despite their commercial availability, peer-reviewed data describing the extent of their field application, overall coverage rates, or strain matching with circulating viruses in Egypt are scarce [1, 55–57]. This variability in vaccine implementation and the limited availability of structured field data underscore the importance of assessing viral circulation in confirmed unvaccinated flocks to better characterize natural infection dynamics within the Egyptian broiler breeder sector.

This study aimed to assess the exposure and molecular prevalence of aMPV in unvaccinated broiler-breeder flocks across multiple Egyptian governorates, and to identify the circulating aMPV subtypes. This information helps provid an integrated epidemiological dataset to support targeted biosecurity and vaccination strategies.

## Materials and methods

### Study design and ethical statement

A cross-sectional study was conducted to investigate the dynamics of aMPV infection in unvaccinated commercial broiler breeder flocks in Egypt during 2024–2025. The study combined serological and molecular surveillance to: (i) determine the prevalence and distribution of aMPV-specific antibodies, (ii) identify the circulating subtypes and (iii) assess age-related seroconversion and cumulative exposure. All procedures of animal handling and sample collection were approved by the Institutional Animal Care and Use Committee (IACUC), Faculty of Veterinary Medicine, Cairo University (Approval No. Vet CU-301220251281).

### Study area and sample collection

During the study period, a multi-stage stratified sampling design was implemented, twelve governorates were included and grouped into three major poultry production zones based on established geographic distribution and production density patterns. The Delta zone (Beheira, Kafr El-Sheikh, Gharbia, Sharqia, Qalyubia, and Menoufia) represents the primary and most densely populated breeder production belt in northern Egypt. Middle Egypt (Giza, Fayoum, and Beni Suef) constitutes a secondary high-density production region. Upper Egypt (Minya, Asyut, and Luxor) includes more geographically dispersed but commercially active breeder operations. This zonal classification allowed structured regional comparison while reflecting ecological and production heterogeneity.

A total of 60 commercial broiler breeder flocks (five flocks per governorate) were enrolled. Within each flock, 50 serum samples were collected at 16 weeks (rearing phase) and 35 weeks (production phase) to assess age-related serological dynamics. All included flocks were confirmed to be unvaccinated against aMPV. Vaccination status was verified through review of official farm vaccination records, consultation with attending veterinarians, and examination of vaccine procurement documentation. Only flocks with documented absence of aMPV vaccination throughout both rearing and production phases were eligible for inclusion. Although mild respiratory signs were observed in a subset of birds during the rearing phase and minor reductions in egg production were reported in some flocks during the production phase, sampling included both clinically affected and apparently healthy birds. This approach was adopted to provide an integrated flock-level assessment of natural aMPV exposure rather than focusing solely on symptomatic individuals. A detailed summary of flock distribution, sampling numbers, and recorded clinical observations is provided in Supplementary Table 1 (Table S1).

For molecular detection, 800 tracheal swabs were collected (10–20 tracheal swabs per flock) during the production period (35 weeks of age). This included 100 swabs per governorate, except for Giza and Fayoum (40 each) and Beni Suef, Minya, Asyut, and Luxor (30 each). Sterile polyester-tipped swabs were used to collect specimens, which were then immediately placed into 3 mL of viral transport medium (VTM) comprised of Anderson’s modified Hanks Balanced Salt Solution (HBSS) supplemented with 2% heat-inactivated fetal bovine serum (FBS), 100 µg/mL gentamicin, and 0.5 µg/mL amphotericin B (Sigma-Aldrich). Samples were maintained at 2–8 °C in insulated ice boxes during transport. Upon arrival at the Virology Laboratory, Faculty of Veterinary Medicine, Cairo University, swab suspensions were vortexed, clarified by brief centrifugation, aliquoted, and stored at − 80 °C until use in molecular analysis.

### Serum preparation and serological analysis

Approximately 1.5 mL of blood was aseptically collected from the wing vein of randomly selected birds and transferred into sterile tubes. Blood was allowed to clot at room temperature and centrifuged at 1000 × g for 10 min. The resultant sera were transferred into labeled Eppendorf tubes, identifying the flock, governorate, age, and collection date, and stored at − 20 °C until testing. Samples showing hemolysis or contamination were excluded from analysis.

Serum samples were analyzed using a commercial indirect ELISA kit (IDVET® Laboratories, Grabels, France) for the detection of aMPV subtypes A and B. Samples were diluted 1:500 and incubated in 96-well microtiter plates pre-coated with aMPV antigens. After incubation and washing, anti-chicken horseradish peroxidase (HRP) conjugate was added, followed by TMB substrate for color development. Each ELISA plate included duplicate wells of the manufacturer-provided control sera: A1 and B1 for the negative control and C1 and D1 for the positive control. Controls were added undiluted according to the kit instructions. The negative and positive controls were used to determine the background optical density (ODNC), calculate S/P ratios, and validate plate performance. Optical density (OD) was measured at 450 nm using a Thermo Scientific™ Multiskan FC™ microplate photometer, as previously described [28, 58]. The sample-to-positive (S/P) ratio was calculated as follows: (Sample OD – NC OD)/(PC OD – NC OD).

Antibody titers were calculated as Log10(Titer) = 1.09 Log10(S/P) + 3.360. The assay was validated if the mean OD_PC_ was greater than 0.250 and the OD_PC_/OD_NC_ ratio was greater than 3. Although the manufacturer recommends a cutoff of S/P > 0.2 (titer > 396) for seropositivity, application of this threshold resulted in an high seropositivity rate in the studied population. Therefore, final serostatus classification was based on a data-driven cutoff derived from distributional analysis of ELISA absorbance values.

### RNA extraction and molecular detection by RT-qPCR

For each flock, every 10 tracheal swabs were pooled, resulting in a total of 80 pools. Viral RNA was extracted from 200 µL of pooled tracheal swabs using QIAamp Viral Mini Kit (Qiagen, Hilden, Germany), according to the manufacturer’s instructions. All procedures were conducted under RNase-free conditions, and eluted RNA was stored at – 80 ºC for downstream processing. All RNA samples were screened for *Mycoplasma gallisepticum*, *Mycoplasma synoviae*, *Ornithobacterium rhinotracheale* (ORT), Newcastle disease virus (NDV), Infectious Bronchitis Virus (IBV), and avian influenza virus (subtypes H5 and H9) using routine RT-PCR assays.

Molecular detection of aMPV was performed using Kylt® aMPV A&B real-time RT-PCR kit (AniCon Labor GmbH, Emstek, Germany) on AB 7500 Fast Real-Time PCR system (Thermo Fisher Scientific, Foster City, CA). The assay simultaneously targeted the G gene of subtypes A and B using specific primers and TaqMan probes labeled with FAM (aMPV-A) and Cy5 (aMPV-B), respectively. Beta-actin specific primers and HEX-labeled probe served as an endogenous control as previously reported [59]. The reaction comprised of 16 µL master mix and 4 µL RNA template, while the thermal profile was adjusted as follows: reverse transcription at 50 °C for 10 min, polymerase activation at 95 °C for 1 min, 42 cycles of denaturation at 95 °C for 10 s, and annealing/extension at 60 °C for 1 min with fluorescence detection in FAM, Cy5, and HEX channels. Positive controls included commercial live vaccines for aMPV subtype A (Poulvac® TRT, Zoetis, Parsippany, NJ) and subtype B (Nemovac®, Merial, Lyon, France), while nuclease-free water served as a negative control. Cycle threshold (Ct) values ≥ 37 were considered negative, and differences > 10 cycles between FAM and Cy5 in the same sample were regarded as questionable.

### Statistical analysis

To determine the optimal ELISA seropositivity threshold, kernel density estimation (KDE) was applied to the distribution of absorbance values. The presence of bimodality was assessed, and the local minimum between density peaks was identified as the empirical cutoff separating seronegative and seropositive populations.

Serological comparisons between production phases were conducted using the Wilcoxon rank-sum test. Subsequent comparisons among governorates and regions were performed using the Kruskal–Wallis test, followed by pairwise post hoc analyses and *p*-value adjustment according to the Bonferroni correction. All statistical analyses and data visualizations were conducted in RStudio (version 2025.09.2) [60] using R programming language (version 4.5.2) [61]. Data are presented as arithmetic means ± standard deviations, and statistical significance was defined as *p* < 0.05.

## Results

### Sero-surveillance of aMPV in broiler breeder flocks

Respiratory manifestations were generally mild and self-limiting, affecting fewer than 6% of birds in Delta flocks and less than 3% in Middle and Upper Egypt. No trivial deviations in mortality or production parameters were observed during the sampling period.

Kernel density analysis of ELISA absorbance values revealed a bimodal distribution, indicating the presence of two distinct serological populations. The local minimum between density peaks (absorbance = 0.555) was identified as the optimal threshold for serostatus classification as shown in Supplementary Fig. S1. Using this classification approach, a high level of aMPV exposure was detected across all surveyed governorates.

Overall, 78.2% of serum samples tested positive for aMPV antibodies, indicating widespread serological evidence of infection at the individual bird level. At the flock level, 100% of sampled broiler breeder flocks were seropositive, confirming that aMPV exposure was present in all investigated production settings. Governorate-level seroprevalence ranged from 64.4% in Luxor to 89% in Giza governorates. Observable geographic variation is provided in Supplementary Table S2.

**Fig. 1:**
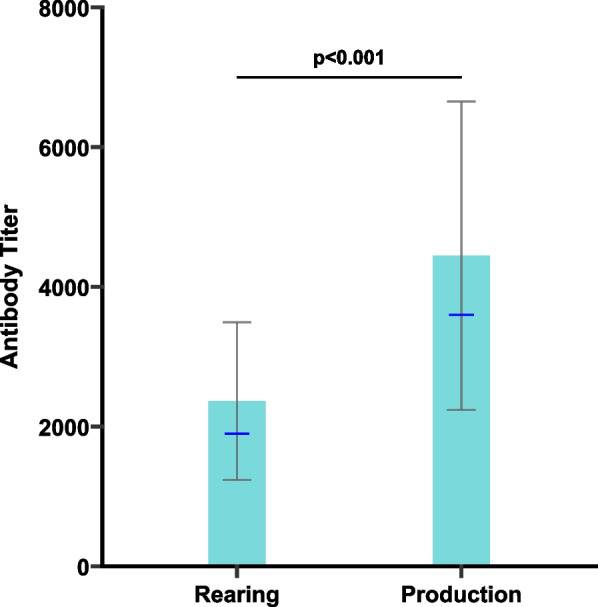
ELISA antibody titers against avian metapneumovirus (aMPV) in unvaccinated broiler breeder flocks during rearing and production phases across Egypt (2024–2025). Differences were assessed using the Wilcoxon rank-sum test. Bars represent the arithmetic mean ± standard deviation, and blue dashes indicate the geometric mean

Antibody titers increased significantly from the rearing phase to the production phase (Fig. 1). Antibody levels at 35-week-old flocks were significantly higher than those observed at 16-week-old flocks (Wilcoxon rank-sum test, *p* < 0.001), consistent with the progressive seroconversion during the production cycle. At 16 weeks, the mean antibody titer was 2,363 ± 1,128 (CV = 47.7%), whereas at 35 weeks the mean titer was 4,426 ± 2,057 (CV = 46.4%).

**Fig. 2:**
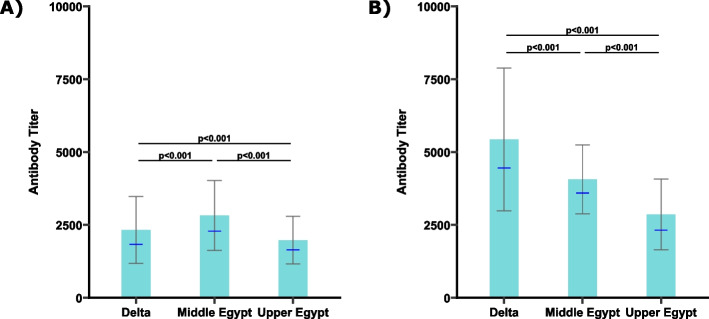
Regional ELISA antibody titers against avian metapneumovirus (aMPV) in non-vaccinated broiler breeder flocks across Egypt (2024–2025). **A** ELISA antibody titers in 16-week-old flocks. **B** ELISA antibody titers in 35-week-old flocks. Differences were assessed using the Kruskal–Wallis test, followed by Bonferroni-corrected pairwise post hoc comparisons. Bars represent the arithmetic mean ± standard deviation, and blue dashes indicate the geometric mean. Statistical significance was set to *p* < 0.05

**Fig. 3:**
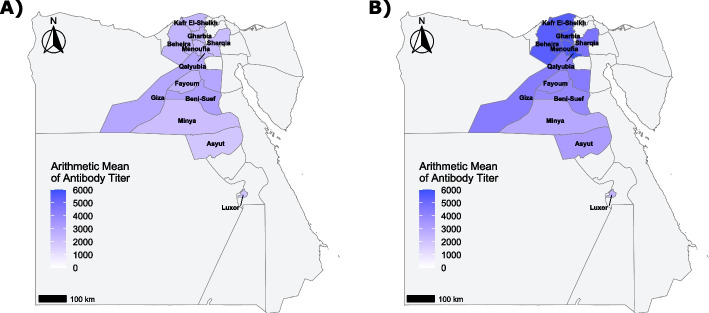
Geographical distribution of ELISA antibody titers against avian metapneumovirus (aMPV) in non-vaccinated broiler breeder flocks across Egyptian governorates (2024–2025). **A** Geographical distribution of ELISA antibody titers in 16-week-old flocks. **B** Geographical distribution of ELISA antibody titers in 35-week-old flocks. Maps are supplemented with a gradient color scale indicating aMPV antibody titer levels. Governorates shown in light grey were not included in the study

At 16 weeks, Middle Egypt region exhibited higher antibody titers compared to Delta and Upper Egypt regions (Fig. 2A). This regional pattern shifted by 35 weeks, when Delta region displayed the highest antibody titers, exceeding those recorded in Middle and Upper Egypt (Fig. 2B). The spatial maps (Fig. 3A and B) revealed a persistent north to south gradient in antibody titers across the production phases, with Upper Egypt flocks consistently exhibiting lower antibody levels.

**Fig. 4:**
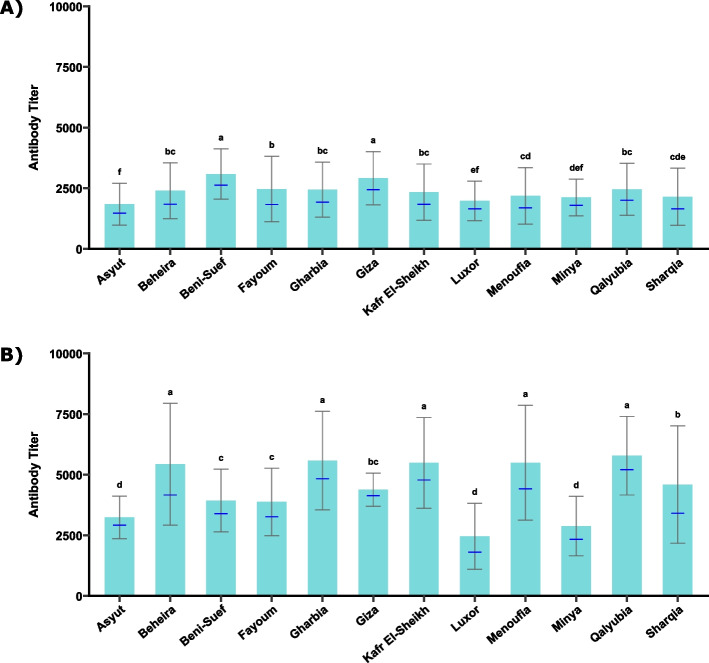
Governorate-level ELISA antibody titers against avian metapneumovirus (aMPV) in non-vaccinated broiler breeder flocks across Egypt (2024–2025). **A** ELISA antibody titers in 16-week-old flocks. **B** ELISA antibody titers in 35-week-old flocks. Differences were assessed using the Kruskal–Wallis test, followed by Bonferroni-corrected pairwise post hoc comparisons. Bars represent the arithmetic mean ± standard deviation, and blue dashes indicate the geometric mean. Governorates that do not share letters differ significantly (*p* < 0.05)

At 16 weeks, governorate-level analysis showed higher antibody titers in Beni Suef and Giza, intermediate titers in Fayoum, Qalyubia, Gharbia, Beheira, and Kafr El-Sheikh, and the lowest titers in Asyut, Minya, and Luxor (Fig. 4A). By 35 weeks, antibody titers increased across all governorates (Fig. 4B). Delta governorates (Qalyubia, Gharbia, Menoufia, Kafr El-Sheikh, and Beheira) formed a high-titer cluster, while Sharqia and Giza exhibited intermediate responses. Lower antibody levels persisted in Beni Suef, Fayoum, and Upper Egypt governorates, confirming sustained geographic heterogeneity throughout the production cycle. The complete dataset of individual raw ELISA absorbance (OD) values and corresponding S/P ratios is provided in Additional file 4.

### Molecular detection and subtyping of aMPV by RT-qPCR

Among the 80 pools of tracheal swabs, 67 (83.75%) tested positive for aMPV RNA. Subtype B was the predominant genotype that was detected solely in 65 pools of samples (81.25%) and co-detected with subtype A in 2 sample pools originated from Beheira governorate (2.5%). No sample pools were exclusively positive for subtype A (Table 1).

**Table 1:** RT-qPCR detection of avian metapneumovirus (aMPV) subtypes in pooled tracheal swabs from broiler breeder flocks in Egypt (2024–2025)

Governorate	Sample Pools (n)	Subtype B positive pools (n)	A + B co-detection (n)	PCR-negative pools (n)
Beheira	10	7	2	1
Kafr El-Sheikh	10	9	0	1
Gharbia	10	9	0	1
Sharqia	10	8	0	2
Qalyubia	10	9	0	1
Menoufia	10	9	0	1
Giza	4	3	0	1
Fayoum	4	3	0	1
Minya	3	2	0	1
Beni Suef	3	2	0	1
Asyut	3	2	0	1
Luxor	3	2	0	1
Total	80	65	2	13

**Fig. 5:**
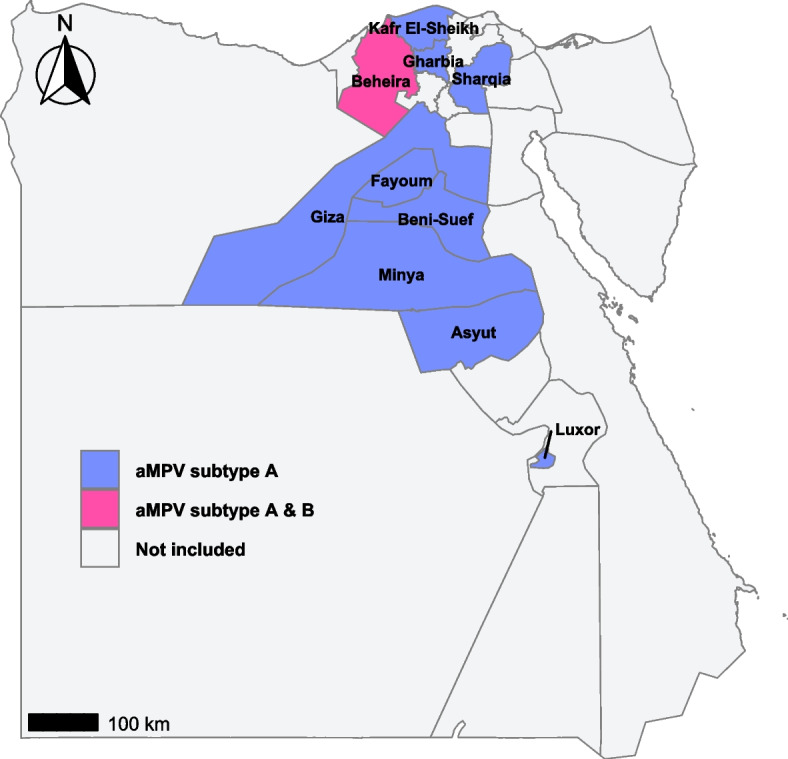
Geographical distribution of avian metapneumovirus (aMPV) subtypes in non-vaccinated broiler breeder flocks across Egyptian governorates (2024–2025). Data are derived from RT-qPCR–positive pooled samples

Positive samples were detected in all Delta, Middle Egypt, and Upper Egypt governorates (Fig. 5), indicating a broad geographic distribution of aMPV. Subtype B was identified in all governorates. High detection rates of subtype B were observed in Delta governorates (80–90%) and slightly decreased toward Middle and Upper Egypt governorates (67.7–75%). The individual raw Ct values generated from the RT-qPCR assays are provided in Additional file 5.

## Discussion

Using an integrated serological and molecular framework, the present study provides a structured assessment of aMPV circulation in unvaccinated broiler breeder flocks across Egypt. By combining dual-age antibody profiling with subtype-specific RT-qPCR targeting the G gene of subtypes A and B, we evaluate both cumulative exposure and concurrent viral detection across multiple governorates, linking immunological evidence with active infection.

The uniformly high seroprevalence (78.2%) observed across all examined flocks indicates widespread exposure within breeder populations. Given that all flocks were confirmed unvaccinated, the detected antibodies are most consistent with natural field infection. Comparable levels of seropositivity have been reported in breeder flocks in Bangladesh (53.3% overall; > 72% in broiler breeders), Iran (93.2%), and South Korea (73.1% overall; 97.3% in broilers and 67.5% in layers) [12, 13, 23, 26], supporting the view that aMPV regional exposure is well established in commercial poultry systems across diverse production settings placing the present findings within a broader epidemiological context.

Interpretation of these findings should consider that ELISA measures exposure rather than functional neutralizing activity. Nevertheless, the commercial indirect ELISA is validated for epidemiological surveillance and is widely applied in aMPV investigations [28, 31]. The high seropositivity observed was supported by concurrent detection of aMPV RNA in pooled tracheal swabs from the same flocks, confirming active viral circulation and reducing the likelihood of non-specific reactivity. Thus, serological and molecular data converge to indicate the ongoing circulation of aMPV subtype A and B.

Molecular analysis demonstrated clear predominance of subtype B, with subtype A not detected as a sole circulating subtype and co-detection of A and B limited to Beheira governorate. This subtype distribution is consistent with reports from Morocco, Tunisia, Algeria, Jordan, and Turkey [14, 15, 38, 39], where subtype B has also been frequently identified. The restricted co-detection observed in Beheira may reflect localized subtype overlap within dense breeder production systems, a pattern that has been described for other avian respiratory pathogens like avian influenza in large commercial populations [62]. However, broader genomic data are required to determine whether this represents stable co-circulation or transient introduction events.

Age-related differences in antibody titers provide additional epidemiological insight. Titers were higher at 35 weeks than at 16 weeks, consistent with progressive exposure or immune boosting over the production cycle, a pattern also described for other respiratory viruses such as infectious bronchitis virus [63]. Variation in titers among flocks and governorates likely reflects heterogeneity in exposure pressure, management practices, and biosecurity conditions as documented for other avian viral diseases such as Marek’s disease and avian leukosis [64], underscoring the multifactorial nature of respiratory virus transmission in breeder operations.

The spatial shift of antibody intensity between production stages further illustrates the interaction between ecological and production-related factors. At 16 weeks of age, higher mean antibody titers were observed in Beni Suef, Giza, and Fayoum governorates. In the surveyed governorates, the sampling was conducted in November 2024, coinciding with the migratory season of wild birds in addition. Furthermore, the proximity of backyard and small-scale breeder operations to wetlands and irrigation canals may increase the risk of indirect pathogen transmission from wild birds [65].

By contrast, the predominance of higher antibody titers in Nile Delta governorates at 35 weeks of age likely reflects sustained within-sector transmission. The Nile Delta is characterized by high poultry density, large flock sizes, and frequent movement of personnel and equipment; conditions known to facilitate viral persistence and spread [47, 66]. Similar density-driven amplification of avian pathogens has been described in North African and Middle Eastern production systems [39, 47–49]. Although causal relationships cannot be established in a cross-sectional design, the observed distribution is consistent with production-stage–related differences in exposure dynamics.

Although the flocks included were unvaccinated, indirect exposure to vaccine-derived viruses from neighboring operations cannot be entirely excluded. Vaccine-derived reversion events have been documented in Europe, including a subtype A aMPV vaccine strain associated with clinical disease in turkeys despite the absence of recent vaccination of affected farms [67, 68].

The subtype-specific RT-qPCR applied does not distinguish field from vaccine strains, and molecular discrimination requires sequence-based analyses [32]. At present, no evidence within this dataset directly indicates vaccine origin. Sequencing of representative high-viral-load samples is ongoing and will allow more precise phylogenetic positioning of circulating strains.

Several limitations should be acknowledged. The cross-sectional design restricts inference regarding transmission directionality and temporal dynamics. In addition, absence of full-length gene or whole-genome sequencing limits resolution for strain differentiation and evolutionary analysis. The use of pooled tracheal swab samples for RT-qPCR analysis may reduce analytical sensitivity in cases of low viral load due to dilution effects. However, pooling is widely accepted in large-scale molecular surveillance programs for avian respiratory viruses because it enables efficient flock-level screening while preserving laboratory resources, and positive pools can be subsequently resolved through individual sample testing when required. Longitudinal surveillance incorporating genomic characterization and targeted investigation at the poultry–wildlife interface will be necessary to clarify introduction pathways and maintenance mechanisms. Further evaluation of potential vertical transmission, as suggested in previous studies [69, 70], is also warranted to better define the epidemiological role of breeder flocks.

## Conclusion

This study demonstrates widespread circulation of aMPV in unvaccinated broiler breeder flocks across surveyed Egyptian governorates, with clear predominance of subtype B. The combined serological and molecular evidence confirms ongoing infection and reveals regional and age-associated variability consistent with differences in production systems. These findings highlight the importance of integrated surveillance and further genomic characterization to better define aMPV epidemiology and support targeted control strategies in breeder populations.

## Supplementary Information

Additional file 1: Table S1. Flock Distribution, Sample Sizes, and Clinical Observations of Studied Broiler Breeder Flocks.

Additional file 2: Table S2. Governorate-level seroprevalence of aMPV antibodies among surveyed broiler breeder flocks in Egypt (2024–2025).

Additional file 3: Figure S1. Histogram of ELISA absorbance (OD) values for all serum samples with an overlaid kernel density curve and cutoff values, providing a visual overview of the serological dataset.

Additional file 4. Raw ELISA dataset containing individual sample-level results. The file contains the following variables: Governorate, Flock ID, Sample ID, Age, Abs (optical density), S/P ratio, Antibody Titer, and Status.

Additional file 5. Individual RT-qPCR cycle threshold (Ct) values obtained from pooled tracheal swab samples analyzed for aMPV detection. The file includes sample identification, governorate, subtype-specific assay results, and Ct values.

## Data Availability

The datasets generated and/or analyzed during the current study are included within the manuscript. Additional data is available from the corresponding author upon reasonable request.

## Abbreviations

aMPV: Avian metapneumovirus
SHS: Swollen Head Syndrome
RT-qPCR: Real-time reverse transcription quantitative polymerase chain reaction
RNA: Ribonucleic acid
ELISA: Enzyme-linked immunosorbent assay
N: Nucleoprotein
P: Phosphoprotein
M: Matrix protein
M2: Matrix protein 2
F: Fusion protein
SH: Small hydrophobic protein
G: Attachment glycoprotein
L: Large polymerase protein
IACUC: Institutional Animal Care and Use Committee
VTM: Viral transport medium
HBSS: Hanks’ Balanced Salt Solution
FBS: Fetal bovine serum
OD: Optical density
ODPC: Optical Density of positive control
ODNC: Optical Density of negative control
S/P: Sample-to-positive ratio
Ct: Cycle threshold
NDV: Newcastle disease virus
IBV: Infectious bronchitis virus
AIV: Avian influenza virus
ORT: Ornithobacterium rhinotracheale
MG: Mycoplasma gallisepticum
MS: Mycoplasma synoviae
HRP: Horseradish peroxidase
TMB: 3,3′,5,5′-Tetramethylbenzidine
PCR: Polymerase chain reaction
RNA: Ribonucleic acid
