# Supplementary material for: Serologic and molecular evidence of avian metapneumovirus subtypes A and B in unvaccinated broiler breeder flocks in Egypt (2024–2025)
Source: BMC Vet Res. 2026 May 9;22:273. doi: 10.1186/s12917-026-05459-y (PMC13156869; doi:10.1186/s12917-026-05459-y)
Supplement: Supplementary file 3 — Additional file 3: Figure S1. Histogram of ELISA absorbance (OD) values for all serum samples with an overlaid kernel density curve and cutoff values, providing a visual overview of the serological dataset. [file 12917_2026_5459_MOESM3_ESM.pdf]

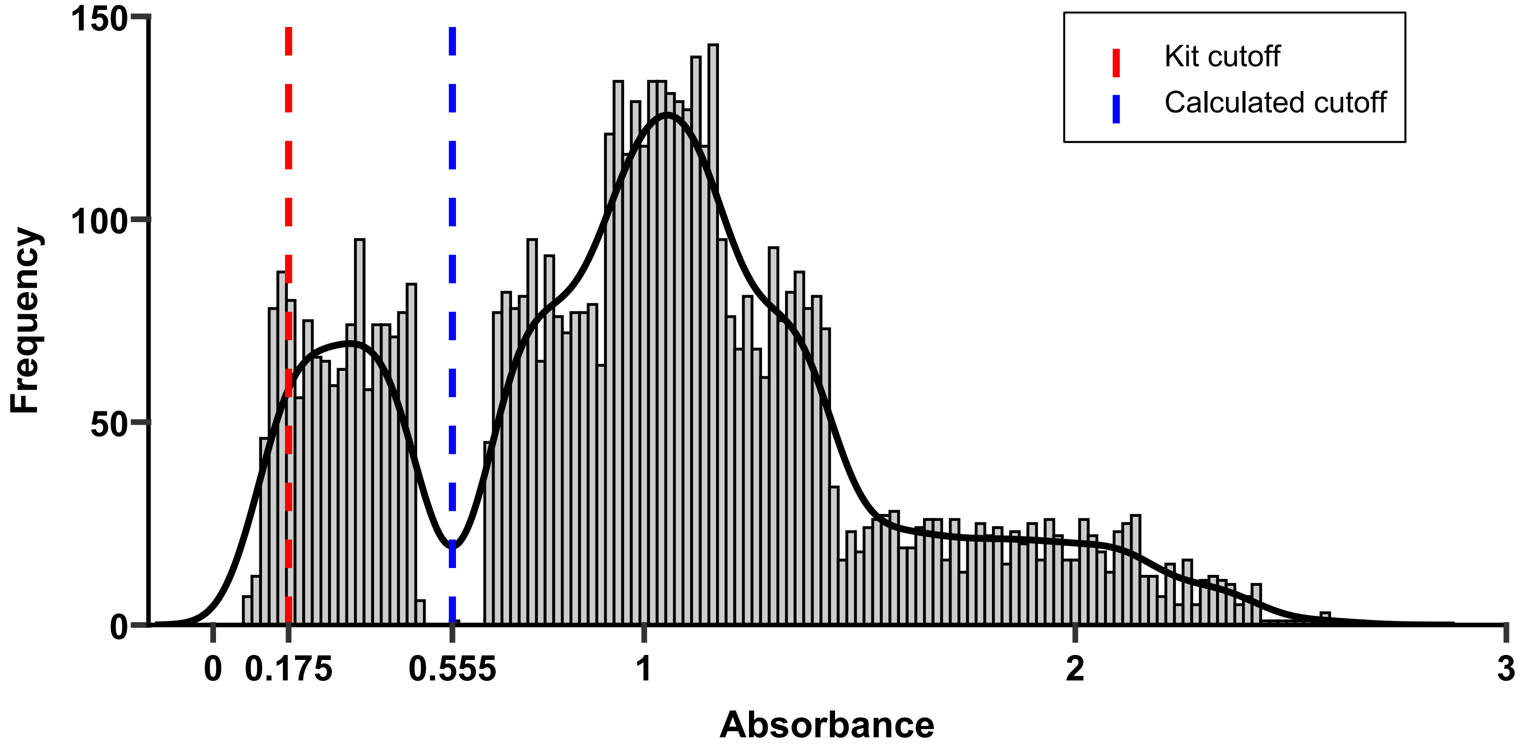

Supplementary Figure 1. Distribution of ELISA absorbance values for all analyzed serum samples. The histogram represents the observed frequency of absorbance values, and the overlaid curve depicts the kernel density estimation of the distribution. The manufacturer-provided (kit) cutoff and the calculated cutoff derived from density valley analysis are indicated by dashed vertical lines.
